# Travel burden for patients with multimorbidity – Proof of concept study in a Dutch tertiary care center^☆^

**DOI:** 10.1016/j.ssmph.2023.101488

**Published:** 2023-08-11

**Authors:** Hidde Dijkstra, Liann I. Weil, Sylvia de Boer, Hubertus P.T.D. Merx, Job N. Doornberg, Barbara C. van Munster

**Affiliations:** a Department of Geriatric Medicine, University Medical Center of Groningen, University of Groningen, the Netherlands; b Department of Orthopaedic Surgery, University Medical Center Groningen, University of Groningen, the Netherlands; c Geodienst, Center for Information Technology, University of Groningen, the Netherlands; d Department of Orthopaedics & Trauma Surgery, Flinders Medical Center and Flinders University, Adelaide, SA, Australia

**Keywords:** Multimorbidity, Travel burden, Video consultation, Geoscience

## Abstract

**Objectives:**

To explore travel burden in patients with multimorbidity and analyze patients with high travel burden, to stimulate actions towards adequate access and (remote) care coordination for these patients.

**Design:**

A retrospective, cross-sectional, explorative proof of concept study.

**Setting and Participants:**

Electronic health record data of all patients who visited our academic hospital in 2017 were used. Patients with a valid 4-digit postal code, aged ≥18 years, had >1 chronic or oncological condition and had >1 outpatient visits with >1 specialties were included.

**Methods:**

Travel burden (hours/year) was calculated as: travel time in hours × number of outpatient visit days per patient in one year × 2. Baseline variables were analyzed using univariate statistics. Patients were stratified into two groups by the median travel burden. The contribution of travel time (dichotomized) and the number of outpatient clinic visits days (dichotomized) to the travel burden was examined with binary logistic regression by adding these variables consecutively to a crude model with age, sex and number of diagnosis. National maps exploring the geographic variation of multimorbidity and travel burden were built. Furthermore, maps showing the distribution of socioeconomic status (SES) and proportion of older age (≥65 years) of the general population were built.

**Results:**

A total of 14 476 patients were included (54.4% female, mean age 57.3 years ([± standard deviation] = ± 16.6 years). Patients travelled an average of 0.42 (± 0.33) hours to the hospital per (one-way) visit with a median travel burden of 3.19 hours/year (interquartile range (IQR) 1.68 – 6.20). Care consumption variables, such as higher number of diagnosis and treating specialties in the outpatient clinic were more frequent in patients with higher travel burden. High travel time showed a higher Odds Ratio (OR = 578 (95% Confidence Interval (CI) = 353 – 947), *p* < 0.01) than having high number of outpatient clinic visit days (OR = 237, 95% CI = 144 – 338), *p* < 0.01) to having a high travel burden in the final regression model.

**Conclusions and implications:**

The geographic representation of patients with multimorbidity and their travel burden varied but coincided locally with lower SES and older age in the general population. Future studies should aim on identifying patients with high travel burden and low SES, creating opportunity for adequate (remote) care coordination.

## Highlights

• Variables related to care consumption were frequently observed in patients with high travel burden and multimorbidity.
• The maps show specific geographic areas of interest with higher travel burden, more multimorbidity, older age and lower socioeconomic status.
• Patients living in such areas would potentially benefit most from eHealth applications, possibly lowering their treatment burden.

## Introduction

The co-occurrence of multiple chronic conditions in the same individual, also known as multimorbidity, became a priority of global health programs (MacMahon and The Academy of Medical Sciences, 2018). In the United States more than one-quarter of adults have multiple chronic conditions and the prevalence of multimorbidity has been increasing (Ansah, 2022; Ford et al., 2013; Ward & Schiller, 2013). Multimorbidity becomes progressively more common with age and is associated with lower health literacy and lower socioeconomic status (SES) (Barnett et al., 2012; Pedersen et al., 2021; Schäfer et al., 2019). Patients with multimorbidity have complex health care needs and multimorbidity has been associated with poorer health care outcomes as well as increased costs and use of care (Chen et al., 2020; Wang et al., 2018).

The disease burden of multimorbidity is high which may be the result of the underlying conditions and the associated health worsening (Chen et al., 2020). Moreover, the high number of primary care consultations, hospital outpatient visits and hospital admissions in patients with multimorbidity contribute to a higher travel burden (Du Vaure et al., 2016; Glynn et al., 2011). Current national health care systems are not designed to adequately meet the care needs for patients with multimorbidity, as services are still fragmented and oriented to managing single diseases instead of complex combinations of conditions (Vogeli et al., 2007). Moreover, as hospitals increasingly offer advanced treatments which are often centralized to tertiary medical centers, patients that need to travel to various medical centers will experience a higher travel burden.

Care coordination and patient-centeredness, two fundamental aspects of multimorbidity care, could potentially decrease the disease related travel burden for patients with multimorbidity (Barbabella et al., 2016). New opportunities enabled by the application and exploitation of eHealth applications, such as virtual visits, could substantially improve care coordination and patient-centeredness. Furthermore, eHealth applications have the potential to improve health care accessibility (Barlow et al., 2007; Clark et al., 2007). In terms of social inclusion and equality, access to health care services can be more difficult for patients with lower SES for a variety of reasons, including unaffordability and residence in deprived areas, typically associated with the presence of multimorbidity (Barnett et al., 2012; Schäfer et al., 2012). Although these groups of patients may benefit most from these eHealth applications, patients with lower SES and older age still show lower adoption of virtual visits (Eberly et al., 2020). Moreover, literature shows that travelling to access healthcare can be particularly burdensome for patients with multimorbidity and their caregivers (Rosbach & Andersen, 2017). The identification of patients with multimorbidity and the highest needs of such applications - by looking at the travel burden – illustrates the need for adequate eHealth adoption in this specific group of patients and would be a first step to implementation.

In this explorative proof of concept study, we will focus on the travel burden of patients with multimorbidity at a Tertiary Academic Care Center in the Netherlands. This hospital provides regular hospital care combined with tertiary highly specialized care. Since the number of academic hospitals in the Netherlands is limited, patients may experience a greater travel burden relative to patients receiving general hospital care in a regional hospital. The aims of this study are (1) to evaluate the travel burden associated with multimorbidity, (2) to explore the patient population with higher travel burden and (3) to gain insight in the geographical distribution of patients with multimorbidity and their travel burden using registry data.

## Methods

### Study design, the Dutch postal coding system and study population

To improve our understanding of the travel burden faced by patients with multimorbidity, a retrospective, cross-sectional cohort study was conducted using an Electronic Health Record (EHR) administrative dataset of all patients who visited the hospital in 2017 (from 01-01–2017 till 31-12-2017).

Postal codes (PC) in the Netherlands consist of four digits followed by two uppercase letters. The first two digits indicate a city or a region, the second two digits and the two letters indicate a range of house numbers, usually on the same street. Postal codes concern areas on the mainland or on a Dutch island or both. Each postal address is uniquely defined by the PC and the house number. Due to privacy reasons, we only used the first four digits of the PC (PC4).

We included patients who met the following criteria:

(1) Patients had a valid PC4 of residence;
(2) Patients were 18 years of age or older on 01-01-2017;
(3) Patients had >1 outpatient visits with >1 specialties in the hospital;
(4) Patients had >1 chronic or oncological conditions, which was registered during the outpatient visit.

The use of the administrative EHR data set has been approved by the Institutional Review Board of the hospital (#20200861, amendment approval number: #107275).

### Data source and study measures

Variables were extracted from the hospital's database, where the EHR data are stored for administrative and billing purposes. In the Netherlands, professionals record Diagnosis-Treatment-Combinations (DTCs) to claim payments. Diagnosis-Treatment-Combinations data include information on the involved specialty per treatment, the diagnosis and the number of care activities such as outpatient visits, emergency department visits and number of hospitalization days. All diagnoses were classified into 152 clinically relevant diagnosis groups using the Dutch Hospital Data - Clinical Classifications Software (DHD-CCS). The diagnoses were classified according to the International Classification of Diseases 10th Revision Procedure Coding System (ICD-10-PCS), which was developed by the Agency for Healthcare Research and Quality (AHRQ) (Supplementary Appendix 1). According to the classification and definitions of the DHD-CCS, all 152 diagnoses were classified into five types of diagnoses (i.e. acute, chronic, elective, oncological and other diagnoses types; Supplementary Appendix 1, Supplementary Table 1.

**Table 1:** Baseline demographic characteristics.

	All patients	Travel burden ≤3.19 hours/year	Travel burden >3.19 hours/year	*P*-value*
	**n = 14 476**	**n = 7294**	**n = 7182**	
Age, mean (±SD) years	57.3 (16.6)	57.4 (17.2)	57.3 (16.1)	0.89
Age (n (%))				<0.01
< 50	4205 (29.0)	2177 (29.8)	2028 (28.2)	
50–65	4619 (31.9)	2229 (30.6)	2390 (33.3)	
65–80	4753 (32.8)	2339 (32.1)	2414 (33.6)	
≥ 80	899 (6.2)	549 (7.5)	350 (4.9)	
Sex, n (%))				<0.01
Female	7872 (54.4)	4069 (55.8)	3803 (53.0)	
Number of diagnoses (n (%))				<0.01
2	6827 (47.2)	4242 (58.2)	2585 (36.0)	
3	4314 (29.8)	2044 (28.0)	2270 (31.6)	
4	1990 (13.7)	700 (9.6)	1290 (18.0)	
5 or more	1345 (9.3)	308 (4.2)	1037 (14.4)	
Number of chronic diagnoses (category) (n (%))				<0.01
0	1601 (11.1)	589 (8.1)	1012 (14.1)	
1	3362 (23.2)	1607 (22.0)	1755 (24.4)	
2	7220 (49.9)	4234 (58.0)	2986 (41.6)	
3 or more	2293 (15.8)	864 (11.8)	1429 (19.9)	
Number of oncological diagnoses (category) (n (%))				<0.01
0	8371 (57.8)	4757 (65.2)	3614 (50.3)	
1	3746 (25.9)	1794 (24.6)	1952 (27.2)	
2 or more	2359 (16.3)	743 (10.2)	1616 (22.5)	
Number of acute diagnoses (category) (n (%))				<0.01
0	11915 (82.3)	6272 (86.0)	5643 (78.6)	
1	2140 (14.8)	886 (12.1)	1254 (17.5)	
2 or more	421 (2.9)	136 (1.9)	285 (4.0)	
Number of elective procedures (category) (n (%))				<0.01
0	11189 (77.3)	6049 (82.9)	5140 (71.6)	
1	2810 (19.4)	1132 (15.5)	1678 (23.4)	
2 or more	477 (3.3)	113 (1.5)	364 (5.1)	
Number of involved specialties (in outpatient clinics (n (%))				<0.01
2	6884 (47.6)	4326 (59.3)	2558 (35.6)	
3	4410 (30.5)	2034 (27.9)	2376 (33.1)	
4	1995 (13.8))	682 (9.4)	1313 (18.3)	
5 or more	1187 (8.2)	252 (3.5)	935 (13.0)	
Number of outpatient clinic visits (category) (n (%))				<0.01
1-3	3921 (27.1)	3306 (45.3)	615 (8.6)	
4–5	4867 (24.8)	2154 (29.5)	1430 (19.9)	
6-9	2388 (25.4)	1381 (18.9)	2290 (31.9)	
10 or more	3300 (22.8	453 (6.2)	2847 (39.6)	
Number of days of outpatient clinic visits (category) (n (%))				<0.01
1-3	4513 (31.2)	3723 (51.0)	790 (11.0)	
4-5	3749 (25.9)	2087 (28.6)	1662 (23.1)	
6-7	2237 (15.5)	811 (11.1)	1426 (19.9)	
8 or more	3977 (27.5)	673 (9.2)	3304 (46.0)	
Mean travel time per hospital visit (in hours) (SD)	0.42 (0.33)	0.26 (0.18)	0.58 (0.37)	<0.01

SD = standard deviation. IQR = Interquartile range.

*Cut-off for significance was 0.05.

Demographic variables (including PC4) and healthcare utilization variables (including type of diagnosis, involved specialties, outpatient clinic visits, outpatient clinic visit days) were collected.

### Calculating travel burden

To create a general dataset of PC4 for the whole continental Netherlands we used two sources; the PC4 overview released by the Central Agency for Statistics (Centraal Bureau voor de Statistiek) and the list of currently existing addresses in 2017 released by the Addresses and Buildings key register (Basisregistratie Adressen en Gebouwen) (Centraal Bureau voor de Statistiek, 2017). To georeference the patient's PC4 as meaningful as possible we first calculated the mean coordinate point of all addresses within each PC4 area in the Netherlands (Centraal Bureau voor de Statistiek, 2017). This was the starting point for the calculation of the travel time to the hospital. The travel times were determined with OpenStreetMap (OSM) (McGowan, 2020). OpenStreetMap is a publicly available dataset and includes all roads of the world. The calculations were performed using pgRouting (an open source route calculating tool) in which Dijkstra's algorithm was used for calculating the shortest route from an origin to a destination (i.e. from the coordinate point to the hospital) (Dijkstra, 1959; McGowan, 2020). The average travel speed by car was used for the calculations, which is slightly below the speed limit.

For island residents, the average travel time with a ferry has been taken into account for the calculations. We added ferry times based on the adverted times for standard ferries on the websites of corporations operating ferries. For the calculations, only patients with PC4 areas which covered geographic areas on the mainland or island were included (i.e. the PC4 area did not cover both; addresses according to the hospital dataset).

The main outcome of interest was the travel burden, which was calculated with the following equation:

Travel time in hours × number of outpatient clinic visit days per patient in one year × 2 = travel burden in hours/year.

The factor 2 was used in the formula because patients generally travel back and forth to the hospital (i.e. two separate trips). After determining the travel burden for each patient, the cohort was stratified into two groups with high and low burden based on the median of the travel burden of the total population.

### The geographic analysis of multimorbidity, travel burden, SES and older age on national maps

First, using the hospital dataset, the absolute geographic distribution of patients with multimorbidity and travel burden were visualized on two separate national maps. The sum of travel burden per PC4 area was used when building the map displaying the absolute geographic distribution of travel burden. Second, the SES of the PC4 areas of the included patients in 2017 was visualized by means of the SES-WOA score (WOA stands for Prosperity, Education and Work) on a national map (using a general dataset). Input for the SES-WOA scores were 1) the national wealth decile of the household, 2) the education level of the household and 3) the household's recent employment history. We used a publicly available list of the average SES-WOA scores per PC4 area of 2017 (Centraal Bureau Statistiek, n.d.). Third, the distribution per PC4 area of the proportion of people ≥65 years of age were visualized. All distributions were visualized per PC4 area and all maps show the location of the hospital as a reference point. For all maps, patients were transformed to point incidents on the map. The PC4 areas were used to “catch” (spatial join) all points and summarize functions have been used for the geographic calculations. All maps were built using AcGIS Pro, an advanced geographic information system that provides extensive capabilities for geospatial data analysis.

### Statistical analysis

All relevant variables were described with univariate statistics. Means, medians, standard deviations (SD) and interquartile ranges (IQR) were calculated (if deemed appropriate) and presented. We conducted the independent *t*-test (normally distributed variables) or Mann Whitney *U* test (non-normally distributed variables) to compare patients with lower and higher travel burden. For categorical variables we performed the Chi-Squared test. A binary logistic regression analyses was performed to explore the relative contribution of high travel time per hospital visit (dichotomized based on median (0.33 hours) and high number of outpatient clinic visit days (dichotomized based on median (5 days) to high travel burden (a composite of travel time and number of hospital visit days, dependent variable). First, a crude model was built with age (in years), sex (male gender), and number of diagnoses as covariates. Thereafter, high travel time and high number of clinic visit days were subsequently added to reach to final model. All reported *P*-values are two-sided with a cut-off value of 0.05 for statistical significance. Statistical analyses were performed using IBM SPSS Version 26.

## Results

### Baseline characteristics and travel burden for patients with multimorbidity

For this study, 14 476 patients met the inclusion criteria (Table 1). The mean age was 57.3 (± 16.6 SD) years and the majority was female (54.4%). Most patients received care for malignancy (43.3%), disease of the circulatory system (26.6%) and disease of the nervous and sensory system (25.3%). The median travel burden was 3.19 hours/year (IQR 1.68 – 6.20) and patients were therefore stratified into two groups: having a travel burden ≤ 3.19 and > 3.19 hours/year. Age groups, sex, number of diagnoses, chronic diagnosis, oncological diagnosis, acute diagnosis, elective procedures, involved specialties in the outpatient clinic and outpatient visit (days) differed significantly among patients with high- and lower travel burden. In the crude model (Table 2), age (OR = 1.0, 95% CI = 1.0–1.0), sex (male gender), (OR = 1.1, 95% CI = 1.0 – 1.2) and number of diagnoses (OR = 1.6, 95% CI = 1.6 – 1.7) were significantly associated with having a high travel burden (p < 0.01). When adjusting for high travel time (model 1), only the number of diagnoses (OR = 2.3, 95% CI = 2.2 – 2.4, p < 0.01) and high travel time (OR = 17.3, 95% CI = 15.7 – 19.0), p < 0.01) were significantly associated with having a high travel burden. In the final model, the number of diagnoses (OR = 1.5, 95% CI = 1.4 – 1.5, p < 0.01), high travel time (OR = 578, 95% CI = 353 – 947), p < 0.01) and high number of outpatient clinic visit days (OR = 237, 95% CI = 144 – 388), p < 0.01) were significantly associated with having a high travel burden. Also, high travel time showed a higher OR for having a high travel burden than high number of patient clinic visit days.

**Table 2:** Crude model and adjusted models for the association between a having a high travel burden ((≥3.19 hour/year, dependent variable), basic variables, high travel time and high number of diagnoses.

	Crude model			Model 1			Final model		
**Variables**	**Beta**	**OR (95% CI)**	*P*-value	Beta	**OR (95% CI)**	***P*-value**	**Beta**	**OR (95% CI)**	***P*-value**
Age in years	0.0	1.0 (1.0–1.0)	<0.01	0.0	1.0 (1.0–1.0)	0.35	0.0	1.0 (1.0–1.0)	0.06
Sex (male gender)	0.1	1.1 (1.0–1.2)	<0.01	0.7	1.1 (1.0–1.2)	0.08	0.0	1.0 (0.9–1.1)	0.94
Number of diagnoses	0.5	1.6 (1.6–1.7)	<0.01	0.8	2.3 (2.2–2.4)	<0.01	0.4	1.5 (1.4–1.5)	<0.01
High travel time per hospital visit (>0.33 h per visit)	–	–	–	2.8	17.3 (15.7–19.0)	<0.01	6.3	578 (353–947)	<0.01
High number of outpatient clinic visit days (≥5)	–	–	–	–	–	–	5.5	237 (144–388)	<0.01

Underlined values indicate a *p-value* ≤ 0.05.

OR = Odds Ratio.

CI = Confidence Interval.

### Geographical distributions

**Fig. 1:**
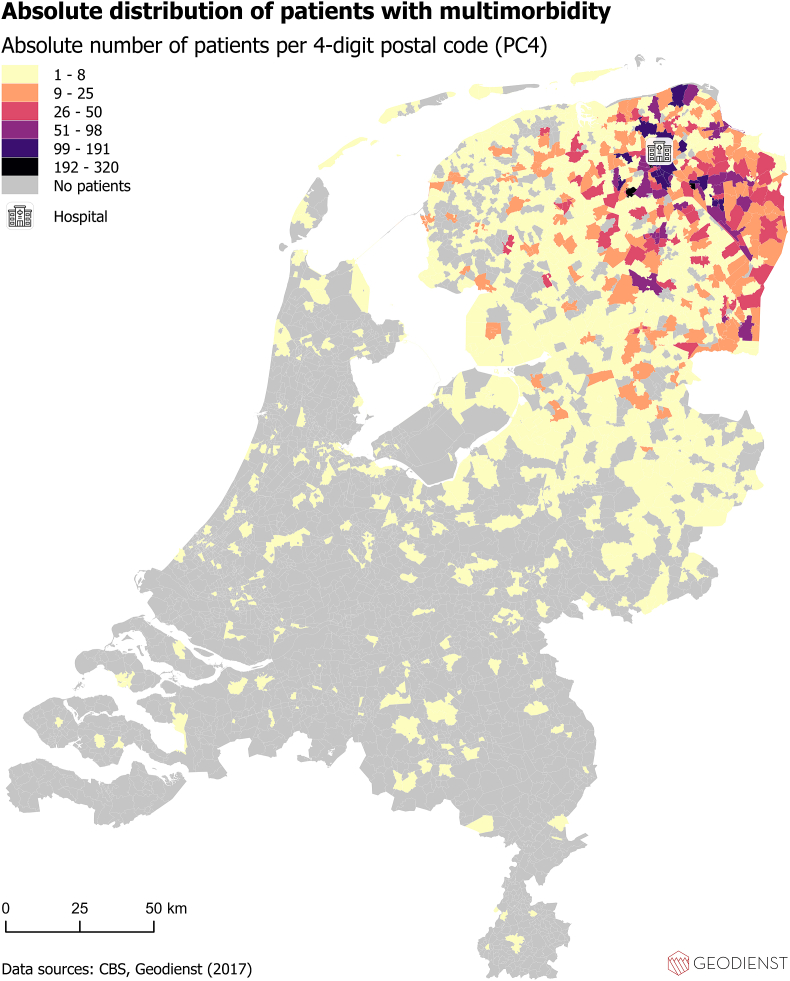
The absolute distribution of patients with multimorbidity per PC4 area in relation to the location of the hospital.

**Fig. 2:**
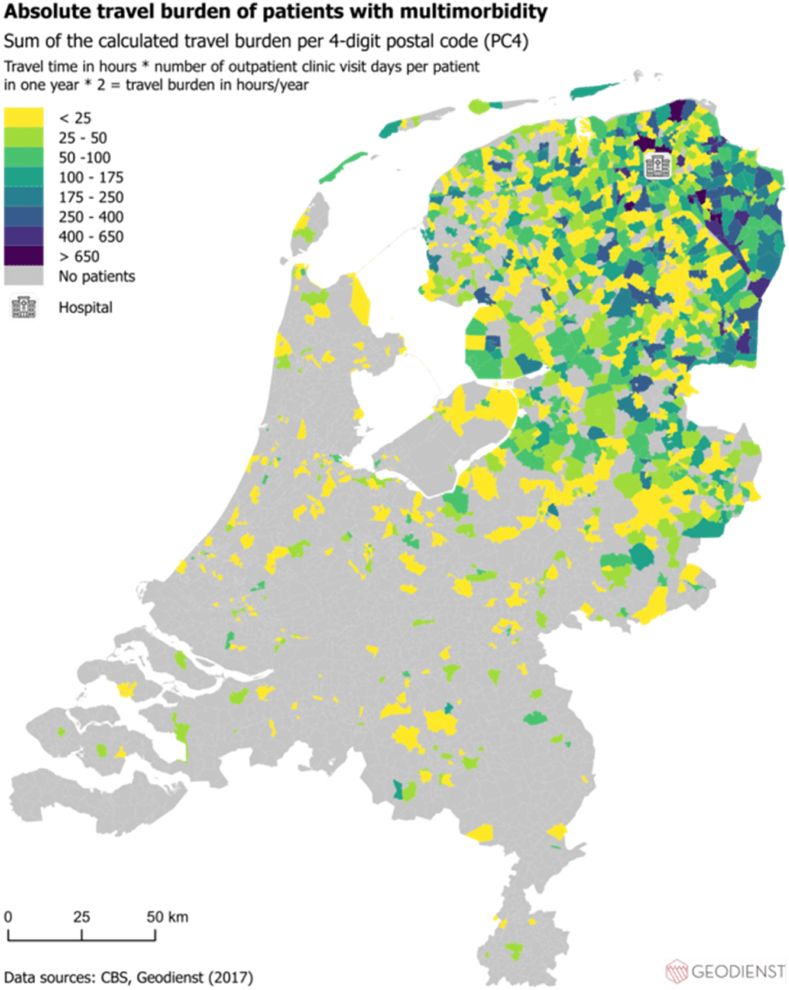
The absolute distribution of the travel burden of patients with multimorbidity per PC4 area in relation to the location of the hospital.

**Fig. 3:**
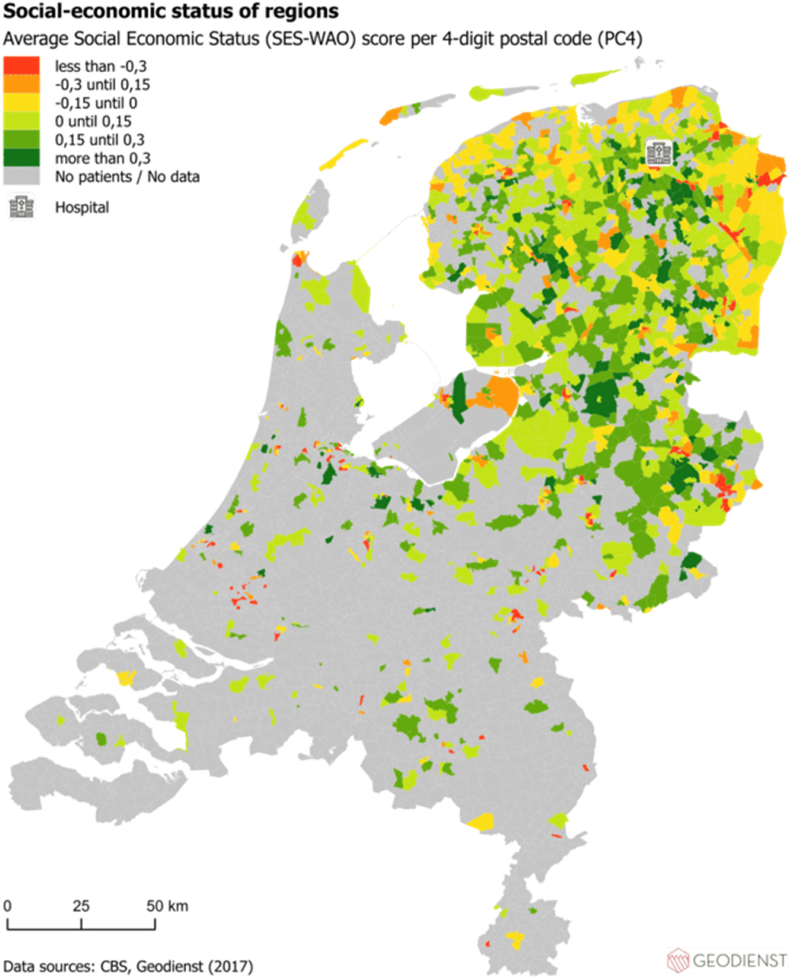
The distribution per PC4 area of socioeconomic status (SES) of the general population who lived in the same PC4 areas as the included patients in 2017 according to the SES-WOA score (WOA stands for Prosperity, Education and Work) in relation to the location of the hospital.

**Fig. 4:**
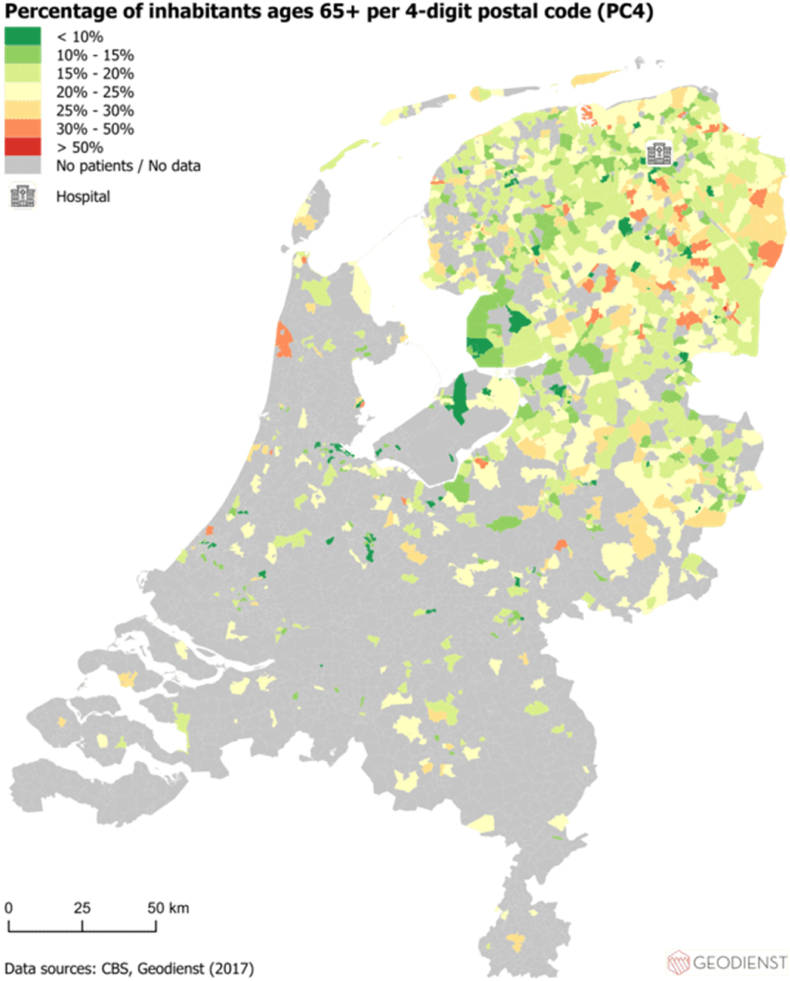
The distribution per PC4 area of the general population ≥65 years of age (as percentage of the total general population) who lived in the same PC4 areas as the included patients in relation to the location of the hospital in 2017.

The absolute distribution of patients with multimorbidity per PC4 area is shown in Fig. 1. The distribution of the absolute travel burden of patients with multimorbidity per PC4 area is shown in Fig. 2. The distribution of SES-WOA scores of the general population who lived in the same PC4 areas as the included patients in 2017 is shown in Fig. 3. The distribution of people of 65 years and older (as percentage of the total population) who lived in the same PC4 areas as the included patients in is shown in Fig. 4. Maps showing the distribution of multimorbidity (relatively), travel burden (relatively), and absolute number of inhabitants per PC4 area (of the PC4 areas of included patients) can be found the Supplementary Appendix 2 (including all maps for readers with color blindness).

## Discussion

Using registry data of a Tertiary Academic Care Center in the Netherlands, we identified specific variables related to care consumption being more frequent in patients with higher travel burden. Travel time to the hospital showed a higher OR than a higher number of involved outpatient clinic visit days to the travel burden in the logistic regression analysis. In addition, the maps built in this study showed specific PC4 areas with higher rates of multimorbidity, travel burden and concomitant lower SES and older age.

In the univariate analysis, we showed that variables related to care consumption, such as a higher number of treating specialties and diagnoses were more frequent in patients with higher travel burden. These findings are as expected: a higher number of diagnoses generally leads to a higher number of hospital visits and therefore higher travel burden. Similar to the findings observed in the univariate analysis, the number of diagnoses remained significant in the final regression model. This may be explained by the interconnectedness of number of diagnoses and outpatient clinic visits. In addition, a higher number of oncologic diagnoses and number of treating specialties were similarly more frequent in patients with higher travel burden. Patients with these variables could potentially benefit from more (centrally) organized (primary) care (as opposed to fragmented, single disease-oriented specialized care). For example, if the number of outpatient clinic visit days for patients is lower, this could lower their overall travel and/or treatment burden. In addition, the logistic regression analysis showed that the amount of time it takes for patients to travel to the hospital has a stronger association with high travel burden than the number of hospital visit days (for outpatient clinic visits). Therefore, travel time might be more important when identifying patients with suspected high travel burden.

Previous research about exploring variables being associated with a higher travel burden in patients with multimorbidity is very limited. Hounkpatin et al. showed that patients with limited health literacy are especially at risk for having a high travel related treatment burden (Hounkpatin et al., 2022). In addition, literature showed that the travel burden for patients with multimorbidity is especially straining to patients with deprived SES and to patients living in remote areas (Rosbach & Andersen, 2017; Sav et al., 2013). Similarly, the maps built in this study show PC4 areas with higher frequency of multimorbidity, concomitant higher travel burden, lower SES and relatively more people of ≥65 years of age (in the general population) in relation to the hospital's location. By eyeballing you see such PC4 areas northern and eastern of the hospital (see Fig. 1, Fig. 2, Fig. 3, Fig. 4). Compared to other PC4 areas with similar multimorbidity representation, by looking for instance south of the location of the hospital, multimorbidity is not always concomitant with lower SES and higher travel burden.

Areas in which multimorbidity, travel burden and lower SES coincide are of particular interest for further research that aims to implement and explore the effect of video-consultations. Such consultations could possibly lower the travel burden and improve access to health care services (Barbabella et al., 2016). Video consultations come with numerous potential advantages such as prevention of unnecessary hospital trips and family members can join consultations without being physically present (Spronk et al., 2022). As such, video consultations may be an alternative solution to overcome barriers associated with an increased travel distance to (tertiary) hospitals. However, the appropriateness of eHealth application may depend on the patient-specific situation. Most research exploring the implementation of *e*Health services is focused around younger, digital-skilled patients with higher SES (Bouabida & Lebouch, 2022), while patients who would benefit most from these implementations are patients with lower SES and higher travel burden.

Our maps show geographic variability in occurrence of multimorbidity (Fig. 1), which is in line with literature exploring the local geographic distribution of multimorbidity. As such, one study identified geographic variability of multimorbidity in New Jersey, United States of America. Interestingly, their study identified specific triads of multimorbidity, such as the colocation of arthritis, hypertension and pulmonary disease (Cromley et al., 2018). Based on a multisource comorbidity score, Corrao et al. (2020) found geographic variability in multimorbidity prevalence in Italy, with differences between northern, central and southern parts of the country. Moreover, Weimann et al. (2016) identified comparable (age adjusted) geographic variability of multimorbidity in South Africa and found a concomitant spatial pattern for socioeconomic status disadvantage. These studies mainly aimed to map the geographic variability of multimorbidity of a certain area. However, they did not focus on the geographic relationship between the geographic variability of multimorbidity and the location of care delivery (i.e. travel burden).

Literature has demonstrated the role of travel burden on health, as it is for example associated with a delay in cancer diagnosis, leading to more advanced disease at diagnosis, inappropriate treatment, worse prognosis and lower quality of life (Ambroggi et al., 2015). Moreover, travel burden seems to be an important part of overall treatment burden for patients with multimorbidity (Hounkpatin et al., 2022; Rosbach & Andersen, 2017). In a time of increasing treatment and travel burden of a growing patient population, it is worth noting that travel burden is something to be taken into account during treatment decision-making. This holds especially for patients with multiple treating specialties, higher number of diagnoses, and large travel distance.

This study includes multiple strengths. First, this is the first study that illustrates the geographical distribution of travel burden of multimorbid patients in the Netherlands using a large administrative EHR dataset from a tertiary medical center. In addition, the presented travel burden is a reliable proxy parameter for the travel burden since patients generally travel to the hospital back and forth via the shortest route at an average speed and taking into consideration the multiple outpatient clinic visits on a single day.

There are some limitations to consider while interpreting the results. First, the results of this study cannot be simply extrapolated to other (larger) countries since the travel distance in the Netherlands is limited due to the small country size. Moreover, only patients >18 years of age with multimorbidity were included. Therefore, the results of this study may not be generalizable to patients with one chronic condition or <18 years of age. Second, for privacy reasons, only the most densely populated area of a PC4 area could be used when calculating and building the maps. This could lead to imprecise representations, especially for patients who live on detached addresses close to the borders of larger PC4 areas. However, we expected that due to the great number of records the deviations were controlled and leveled to average. Third, the travel burden was calculated on the assumption that all patients used private automobiles. Nonetheless, particularly low-income patients may rely on public transportation which may have different travel times or distances than assigned in this study. In addition, it is known that low-income patients more frequently have multimorbidity and these patients more frequently live in deprived areas (Pathirana & Jackson, 2018). Therefore, the travel burden for this group of patients may be underreported in this study. Fourth, when calculating the travel burden only the days of outpatient clinic visits were included. The travel burden of other hospital visitations such as for diagnostic examinations (e.g. blood tests or X-rays) or hospital admissions were not included. Therefore, it can be speculated that the travel burden of the most severe multimorbid patients might have been higher than depicted in this study.

## Conclusion and implications

It is expected that the travel burden of patients with multimorbidity will increase. It is therefore necessary to gain a further understanding of which patients with multimorbidity have a high travel burden. We found variables related to higher care consumption such as a higher number of treating specialties, oncological diagnosis and acute diagnosis being more frequent in patients with a higher travel burden. In addition, in the explorative geographical analysis, we identified local PC4 areas with higher rates of multimorbidity, travel burden, lower SES and older age. Especially this group of patients might benefit most from eHealth applications. Local healthcare initiatives should aim for identifying multimorbid patients with the highest travel burden and low SES, creating a target group for improving both patient-centeredness and care coordination to possibly lower their overall treatment burden.

## Funding sources

This research did not receive any specific grant from funding agencies in the public, commercial or not-for-profit sectors.

## Declaration of competing interest

Each author certifies that he or she has no commercial associations (e.g., consultancies, stock ownership, equity interest, patent/licensing arrangements, etc) that might pose a conflict of interest in connection with the submitted article.

## Data Availability

The authors do not have permission to share data.
