# Supplementary material for: Travel burden for patients with multimorbidity – Proof of concept study in a Dutch tertiary care center
Source: SSM Popul Health. 2023 Aug 11;24:101488. doi: 10.1016/j.ssmph.2023.101488 (PMC10483049; doi:10.1016/j.ssmph.2023.101488)
Supplement: Multimedia component 1 [file mmc1.docx]

**Appendix 1 of**

Travel Burden for Patients with Multimorbidity – a proof of concept study of a Dutch tertiary care center

| **Supplementary Table 1.** Diagnosis groups according to the Dutch Hospital Data-Clinical Classification Software (DHD-CCS). | |
| --- | --- |
| **Diagnosis group** | **Diagnosis type** |
| Abdominal hernia | Elective |
| Abdominal pain | Chronic |
| Acquired foot deformities | Chronic |
| Acute and chronic tonsillitis | Chronic |
| Acute and unspecified renal failure | Acute |
| Acute bronchitis | Acute |
| Acute cerebrovascular disease | Acute |
| Acute myocardial infarction | Acute |
| Adjustment disorders | Chronic |
| Administrative/social admission | Other |
| Alcohol-related disorders | Other |
| Allergic reactions | Chronic |
| Anal and rectal conditions | Elective |
| Anxiety disorders | Chronic |
| Aortic and peripheral arterial embolism or thrombosis | Acute |
| Aortic; peripheral; and visceral artery aneurysms | Acute |
| Appendicitis and other appendiceal conditions | Acute |
| Asthma | Chronic |
| Bacterial infection; unspecified site | Acute |
| Benign neoplasm of uterus | Elective |
| Biliary tract disease | Elective |
| Birth trauma | Other |
| Blindness and vision defects | Chronic |
| Burns | Acute |
| Calculus of urinary tract | Elective |
| Cancer of bladder | Oncologic |
| Cancer of bone and connective tissue | Oncologic |
| Cancer of brain and nervous system | Oncologic |
| Cancer of breast | Oncologic |
| Cancer of bronchus; lung | Oncologic |
| Cancer of cervix | Oncologic |
| Cancer of colon | Oncologic |
| Cancer of esophagus | Oncologic |
| Cancer of head and neck | Oncologic |
| Cancer of kidney and renal pelvis | Oncologic |
| Cancer of liver and intrahepatic bile duct | Oncologic |
| Cancer of other female genital organs | Oncologic |
| Cancer of other GI organs; peritoneum | Oncologic |
| Cancer of other male genital organs | Oncologic |
| Cancer of other urinary organs | Oncologic |
| Cancer of ovary | Oncologic |
| Cancer of pancreas | Oncologic |
| Cancer of prostate | Oncologic |
| Cancer of rectum and anus | Oncologic |
| Cancer of stomach | Oncologic |
| Cancer of testis | Oncologic |
| Cancer of thyroid | Oncologic |
| Cancer of uterus | Oncologic |
| Cancer; other and unspecified primary | Oncologic |
| Cancer; other respiratory and intrathoracic | Oncologic |
| Cardiac and circulatory congenital anomalies | Chronic |
| Cardiac arrest and ventricular fibrillation | Acute |
| Cardiac dysrhythmias | Acute |
| Cataract | Elective |
| Chronic kidney disease | Chronic |
| Chronic obstructive pulmonary disease and bronchiectasis | Chronic |
| Chronic ulcer of skin | Chronic |
| Coagulation and hemorrhagic disorders | Chronic |
| Coma; stupor; and brain damage | Acute |
| Complication of device; implant or graft | Other |
| Complications of surgical procedures or medical care | Acute |
| Conditions associated with dizziness or vertigo | Chronic |
| Conduction disorders | Chronic |
| Congestive heart failure; non-hypertensive | Chronic |
| Contraceptive and procreative management | Elective |
| Coronary atherosclerosis and other heart disease | Chronic |
| Crushing injury or internal injury | Acute |
| Cystic fibrosis | Chronic |
| Deficiency and other anemia | Chronic |
| Delirium dementia and amnestic and other cognitive disorders | Chronic |
| Diabetes mellitus with complications | Chronic |
| Diabetes mellitus without complication | Chronic |
| Diabetes or abnormal glucose tolerance complicating pregnancy; childbirth; or the puerperium | Chronic |
| Digestive congenital anomalies | Acute |
| Diseases of mouth; excluding dental | Elective |
| Diseases of white blood cells | Chronic |
| Disorders of lipid metabolism | Chronic |
| Disorders of teeth and jaw | Other |
| Disorders usually diagnosed in infancy childhood or adolescence | Chronic |
| Diverticulosis and diverticulitis | Chronic |
| Ectopic pregnancy | Other |
| Encephalitis (except that caused by tuberculosis or sexually transmitted disease) | Acute |
| Endometriosis | Chronic |
| Epilepsy; convulsions | Chronic |
| Esophageal disorders | Chronic |
| Essential hypertension | Chronic |
| Female infertility | Elective |
| Fever of unknown origin | Acute |
| Fluid and electrolyte disorders | Other |
| Fracture of lower limb | Acute |
| Fracture of neck of femur (hip) | Acute |
| Fracture of upper limb | Acute |
| Gastritis and duodenitis | Chronic |
| Gastroduodenal ulcer (except hemorrhage) | Elective |
| Gastrointestinal hemorrhage | Chronic |
| Genitourinary congenital anomalies | Chronic |
| Genitourinary symptoms and ill-defined conditions | Chronic |
| Glaucoma | Elective |
| Gout and other crystal arthropathies | Chronic |
| Headache; including migraine | Chronic |
| Heart valve disorders | Elective |
| Hemolytic jaundice and perinatal jaundice | Acute |
| Hemorrhoids | Elective |
| Hepatitis | Chronic |
| HIV infection | Chronic |
| Hodgkin`s disease | Oncologic |
| Hyperplasia of prostate | Chronic |
| Hypertension complicating pregnancy; childbirth and the puerperium | Other |
| Immunity disorders | Chronic |
| Immunizations and screening for infectious disease | Acute |
| Infective arthritis and osteomyelitis (except that caused by tuberculosis or sexually transmitted disease) | Chronic |
| Inflammation; infection of eye (except that caused by tuberculosis or sexually transmitted disease) | Acute |
| Inflammatory conditions of male genital organs | Acute |
| Inflammatory diseases of female pelvic organs | Acute |
| Intestinal infection | Acute |
| Intestinal obstruction without hernia | Acute |
| Intracranial injury | Acute |
| Joint disorders and dislocations; trauma-related | Acute |
| Late effects of cerebrovascular disease | Other |
| Leukemias | Oncologic |
| Liver disease, alcohol-related | Chronic |
| Lung disease due to external agents | Chronic |
| Lymphadenitis | Chronic |
| Malaise and fatigue | Chronic |
| Malignant neoplasm without specification of site | Oncologic |
| Malposition; malpresentation | Elective |
| Medical examination/evaluation | Other |
| Melanomas of skin | Oncologic |
| Meningitis (except that caused by tuberculosis or sexually transmitted disease) | Acute |
| Menopausal disorders | Chronic |
| Menstrual disorders | Chronic |
| Miscellaneous mental health disorders | Chronic |
| Mood disorders | Chronic |
| Multiple myeloma | Oncologic |
| Multiple sclerosis | Chronic |
| Mycoses | Chronic |
| Nausea and vomiting | Chronic |
| Neoplasms of unspecified nature or uncertain behavior | Oncologic |
| Nephritis; nephrosis; renal sclerosis | Chronic |
| Nervous system congenital anomalies | Chronic |
| Non-Hodgkin`s lymphoma | Oncologic |
| Noninfectious gastroenteritis | Acute |
| Nonmalignant breast conditions | Elective |
| Nonspecific chest pain | Acute |
| Nutritional deficiencies | Chronic |
| Occlusion or stenosis of precerebral arteries | Other |
| Open wounds of extremities | Acute |
| Open wounds of head; neck; and trunk | Acute |
| Osteoarthritis | Elective |
| Osteoporosis | Chronic |
| Other acquired deformities | Elective |
| Other aftercare | Other |
| Other and ill-defined cerebrovascular disease | Acute |
| Other and ill-defined heart disease | Chronic |
| Other and unspecified benign neoplasm | Elective |
| Other bone disease and musculoskeletal deformities | Chronic |
| Other circulatory disease | Chronic |
| Other CNS infection and poliomyelitis | Chronic |
| Other complications of birth; puerperium affecting management of mother | Acute |
| Other complications of pregnancy | Elective |
| Other congenital anomalies | Chronic |
| Other connective tissue disease | Chronic |
| Other diseases of bladder and urethra | Acute |
| Other diseases of kidney and ureters | Chronic |
| Other diseases of veins and lymphatics | Chronic |
| Other disorders of stomach and duodenum | Chronic |
| Other ear and sense organ disorders | Chronic |
| Other endocrine disorders | Elective |
| Other eye disorders | Elective |
| Other female genital disorders | Chronic |
| Other fractures | Acute |
| Other gastrointestinal disorders | Chronic |
| Other hematologic conditions | Chronic |
| Other hereditary and degenerative nervous system conditions | Chronic |
| Other infections; including parasitic | Acute |
| Other inflammatory condition of skin | Chronic |
| Other injuries and conditions due to external causes | Acute |
| Other liver diseases | Chronic |
| Other lower respiratory disease | Chronic |
| Other male genital disorders | Elective |
| Other nervous system disorders | Chronic |
| Other non-epithelial cancer of skin | Oncologic |
| Other non-traumatic joint disorders | Chronic |
| Other nutritional; endocrine; and metabolic disorders | Chronic |
| Other perinatal conditions | Elective |
| Other pregnancy and delivery including normal | Elective |
| Other screening for suspected conditions (not mental disorders or infectious disease) | Elective |
| Other skin disorders | Chronic |
| Other upper respiratory disease | Chronic |
| Other upper respiratory infections | Elective |
| Otitis media and related conditions | Acute |
| Pancreatic disorders (not diabetes) | Acute |
| Paralysis | Chronic |
| Parkinson`s disease | Chronic |
| Pathological fracture | Chronic |
| Peri-; endo-; and myocarditis; cardiomyopathy (except that caused by tuberculosis or sexually transmitted disease) | Chronic |
| Peripheral and visceral atherosclerosis | Chronic |
| Peritonitis and intestinal abscess | Acute |
| Phlebitis; thrombophlebitis and thromboembolism | Acute |
| Pleurisy; pneumothorax; pulmonary collapse | Acute |
| Pneumonia (except that caused by tuberculosis or sexually transmitted disease) | Acute |
| Poisoning by nonmedicinal substances | Acute |
| Previous C-section | Other |
| Prolapse of female genital organs | Chronic |
| Pulmonary heart disease | Chronic |
| Regional enteritis and ulcerative colitis | Chronic |
| Rehabilitation care; fitting of prostheses; and adjustment of devices | Other |
| Residual codes; unclassified | Chronic |
| Respiratory distress syndrome | Other |
| Respiratory failure; insufficiency; arrest (adult) | Acute |
| Retinal detachments; defects; vascular occlusion; and retinopathy | Elective |
| Rheumatoid arthritis and related disease | Chronic |
| Schizophrenia and other psychotic disorders | Chronic |
| Screening and history of mental health and substance abuse codes | Other |
| Secondary malignancies | Oncologic |
| Septicemia (except in labor) | Acute |
| Sexually transmitted infections (not HIV or hepatitis) | Acute |
| Sickle cell anemia | Chronic |
| Skin and subcutaneous tissue infections | Acute |
| Skull and face fractures | Acute |
| Spinal cord injury | Acute |
| Spondylosis; intervertebral disc disorders; other back problems | Chronic |
| Sprains and strains | Acute |
| Substance-related disorders | Chronic |
| Superficial injury; contusion | Acute |
| Syncope | Acute |
| Systemic lupus erythematosus and connective tissue disorders | Chronic |
| Thyroid disorders | Chronic |
| Transient cerebral ischemia | Acute |
| Tuberculosis | Chronic |
| Urinary tract infections | Elective |
| Varicose veins of lower extremity | Elective |
| Viral infection | Acute |
