# Supplementary material for: Travel burden for patients with multimorbidity – Proof of concept study in a Dutch tertiary care center
Source: SSM Popul Health. 2023 Aug 11;24:101488. doi: 10.1016/j.ssmph.2023.101488 (PMC10483049; doi:10.1016/j.ssmph.2023.101488)
Supplement: Multimedia component 2 [file mmc2.docx]

**Appendix 2 of**

Travel Burden for Patients with Multimorbidity – a proof of concept study of a Dutch tertiary care center

Part 1: additional maps.

- **Figure A1 (page 2).** The relative distribution of patients with multimorbidity per Postal Code (PC) 4 area in relation to the location of the hospital.
- **Figure A2 (page 3).** The relative distribution of the travel burden of patients with multimorbidity per PC4 area in relation to the location of the hospital. The
- **Figure A3 (page 4)**. The absolute distribution of the general population living in the PC4 areas of the patients.

Part 2: all maps for people with color blindness.

- **Figure A4 (page 5).** The absolute distribution of patients with multimorbidity per PC4 area in relation to the location of the hospital.
- **Figure A5 (page 6).** The absolute distribution of the travel burden of patients with multimorbidity per PC4 area in relation to the location of the hospital.
- **Figure A6 (page 7).** The distribution per PC4 area of socioeconomic status (SES) of the general population who lived in the same PC4 areas as the included patients in 2017 according to the SES-WOA score (WOA stands for Prosperity, Education and Work) in relation to the location of the hospital.
- **Figure A7 (page 8).** The distribution per PC4 area of the general population ≥ 65 years of age (as percentage of the total general population) who lived in the same PC4 areas as the included patients in relation to the location of the hospital in 2017.
- **Figure A8 (page 9).** The relative distribution of patients with multimorbidity per PC4 area in relation to the location of the hospital.
- **Figure A9 (page 10).** The relative distribution of the travel burden of patients with multimorbidity per PC4 area in relation to the location of the hospital. The
- **Figure A10 (page 11)**. The absolute distribution of the general population living in the PC4 areas of the patients.

**
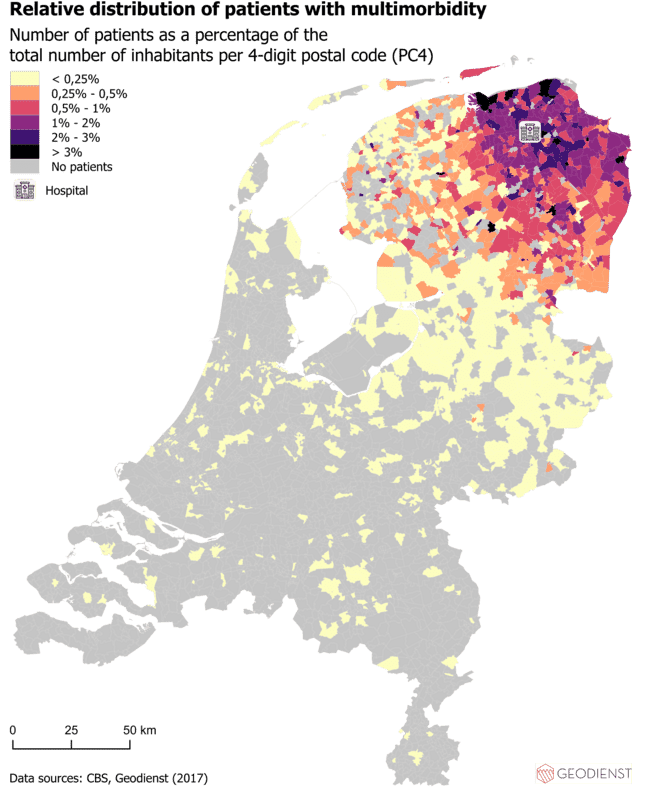
**

**Figure A1.** The relative distribution of patients with multimorbidity per PC4 area in relation to the location of the hospital. The total number of patients with multimorbidity was divided by the total number of inhabitants per PC4 area for the calculation.

**
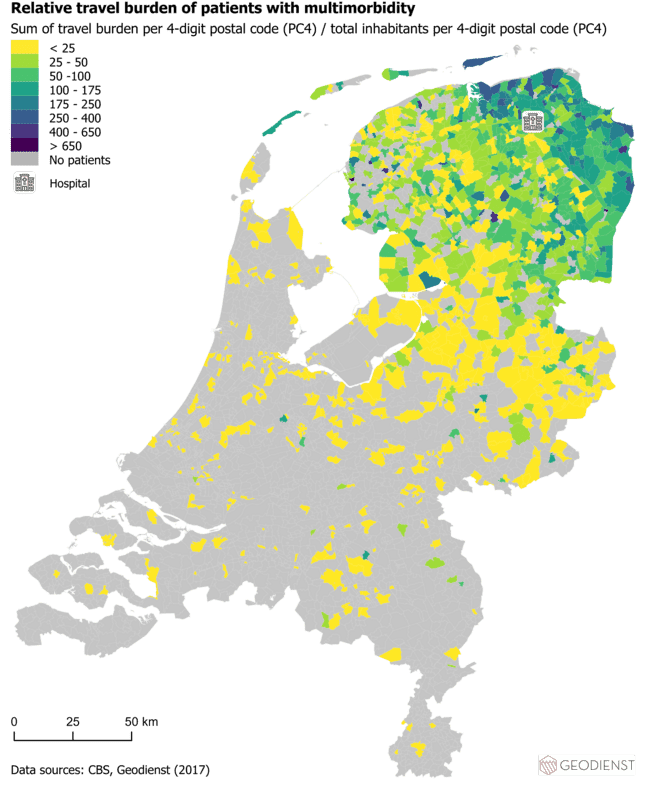
**

**Figure A2.** The relative distribution of the travel burden of patients with multimorbidity per PC4 area in relation to the location of the hospital. The sum of the travel burden per PC4 area was divided by the total number of inhabitants per PC4 area for the calculation.

**
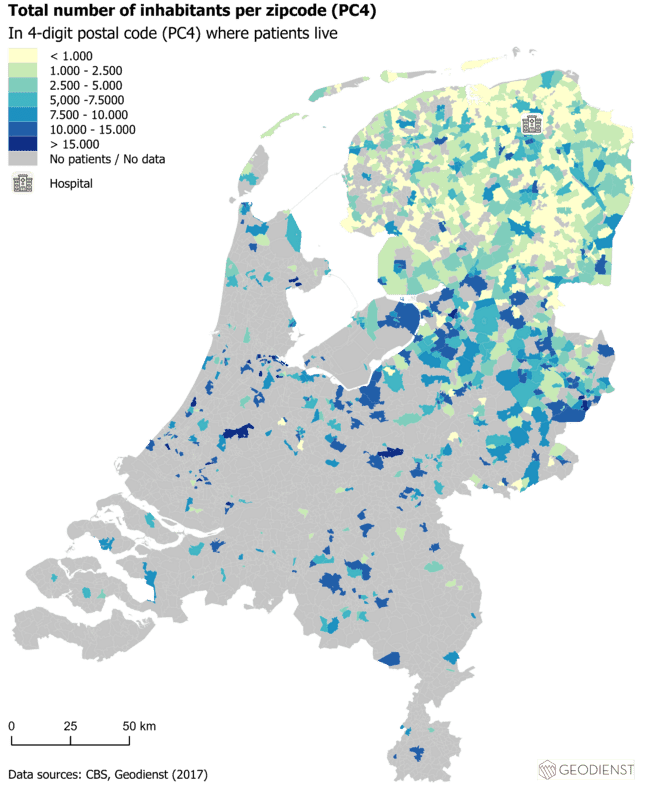
**

**Figure A3.** The absolute distribution of the general population living in the PC4 areas of the patients.


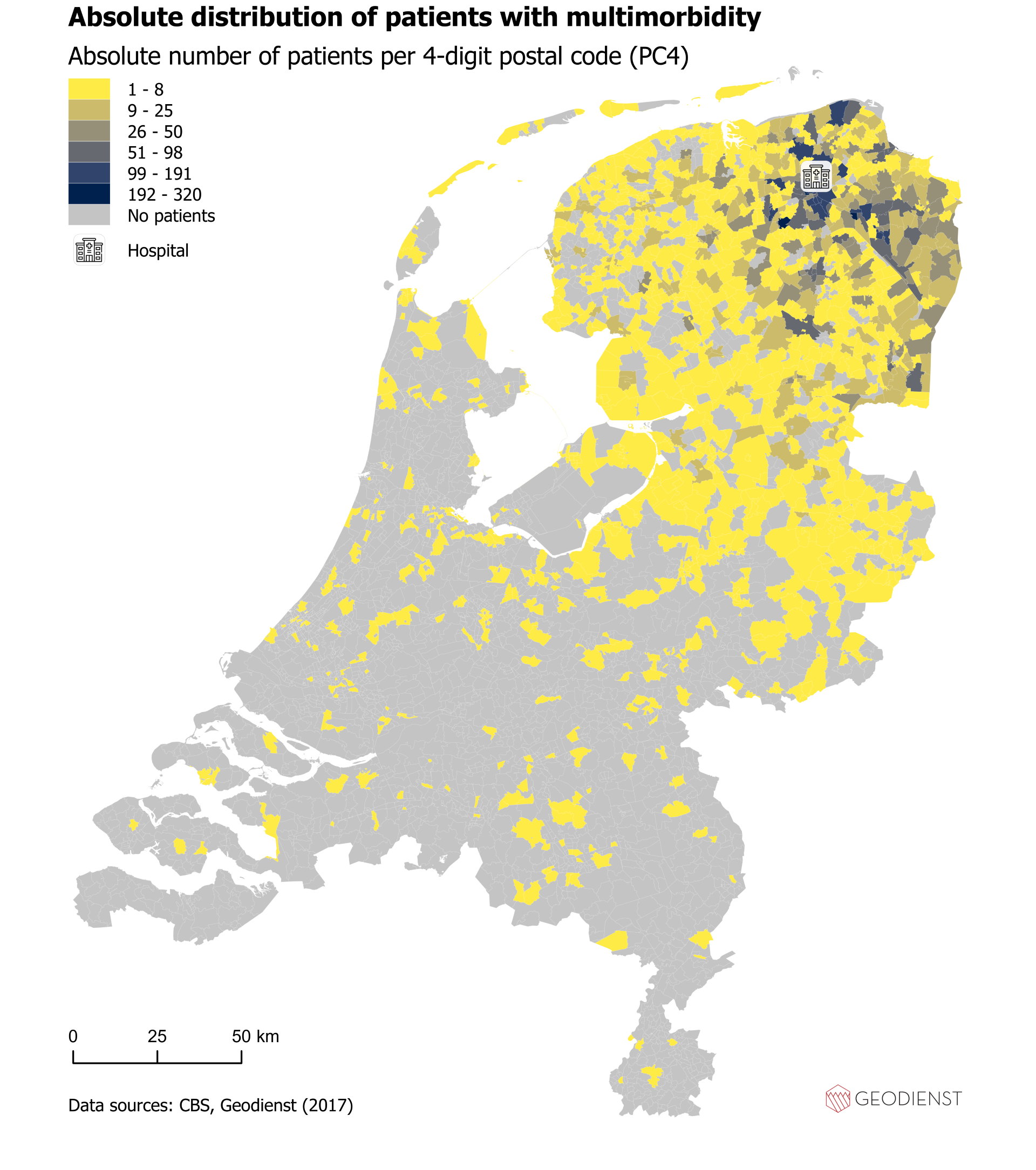


**Figure A4.** The absolute distribution of patients with multimorbidity per PC4 area in relation to the location of the hospital.


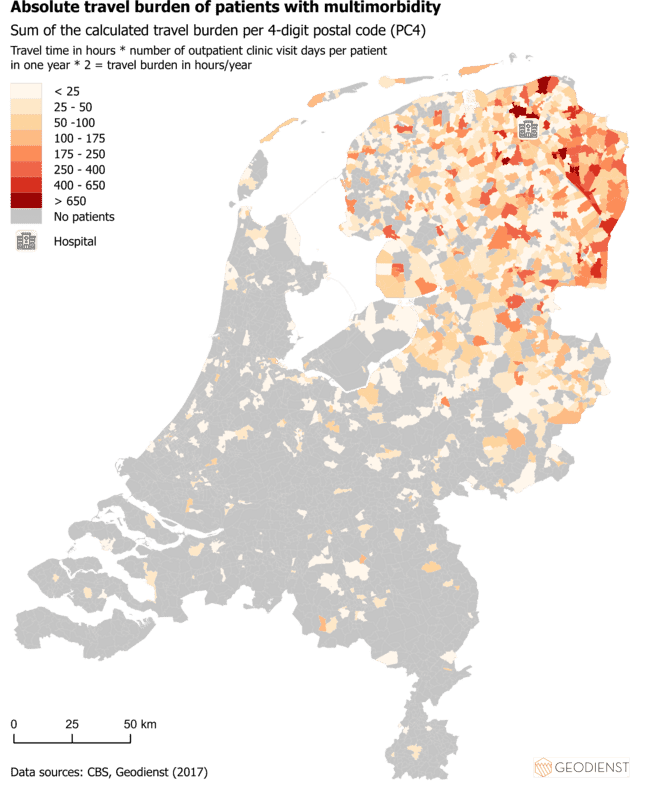


**Figure A5.** The absolute distribution of the travel burden of patients with multimorbidity per PC4 area in relation to the location of the hospital.


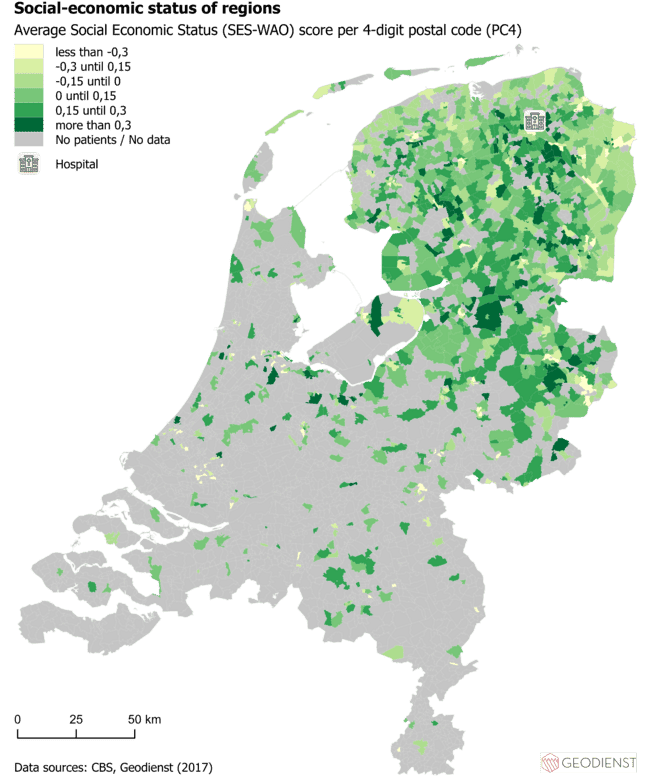


**Figure A6.** The distribution per PC4 area of socioeconomic status (SES) of the general population who lived in the same PC4 areas as the included patients in 2017 according to the SES-WOA score (WOA stands for Prosperity, Education and Work) in relation to the location of the hospital.


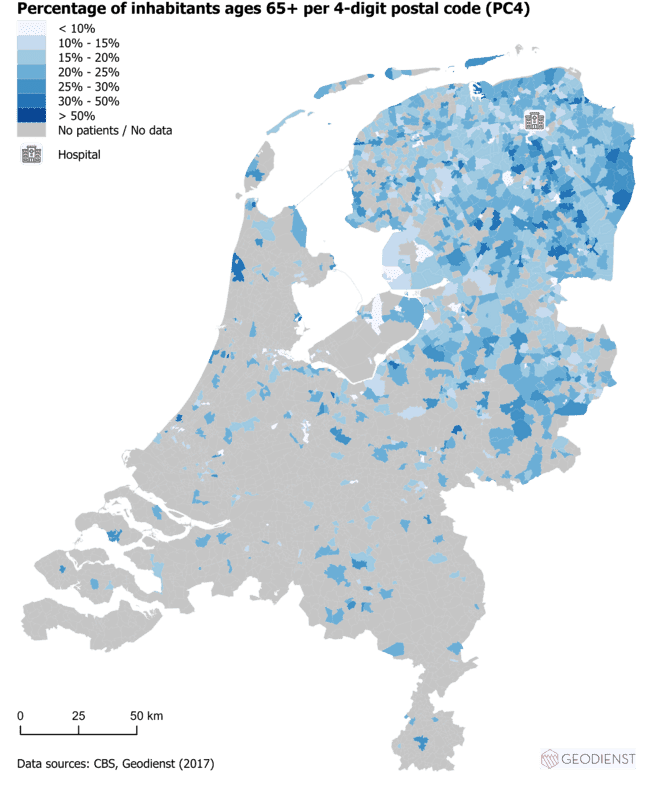


**Figure A7.** The distribution per PC4 area of the general population ≥ 65 years of age (as percentage of the total general population) who lived in the same PC4 areas as the included patients in relation to the location of the hospital in 2017.


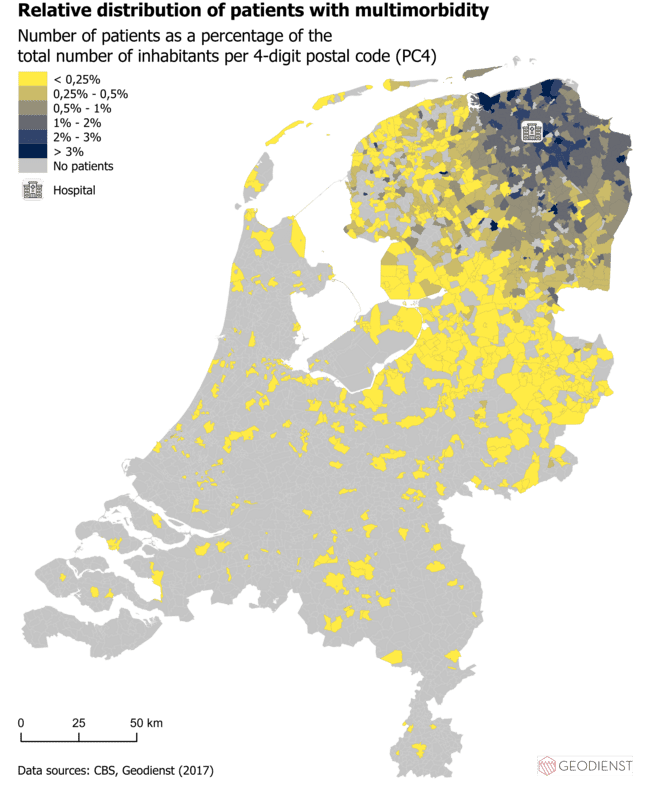


**Figure A8.** The relative distribution of patients with multimorbidity per PC4 area in relation to the location of the hospital. The total number of patients with multimorbidity was divided by the total number of inhabitants per PC4 area for the calculation.


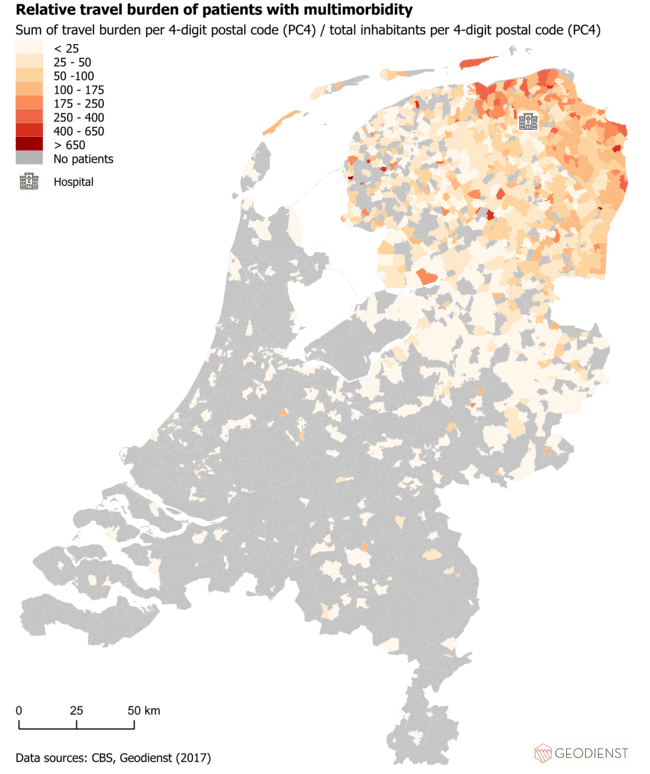


**Figure A9.** The relative distribution of the travel burden of patients with multimorbidity per PC4 area in relation to the location of the hospital. The sum of the travel burden per PC4 area was divided by the total number of inhabitants per PC4 area for the calculation.


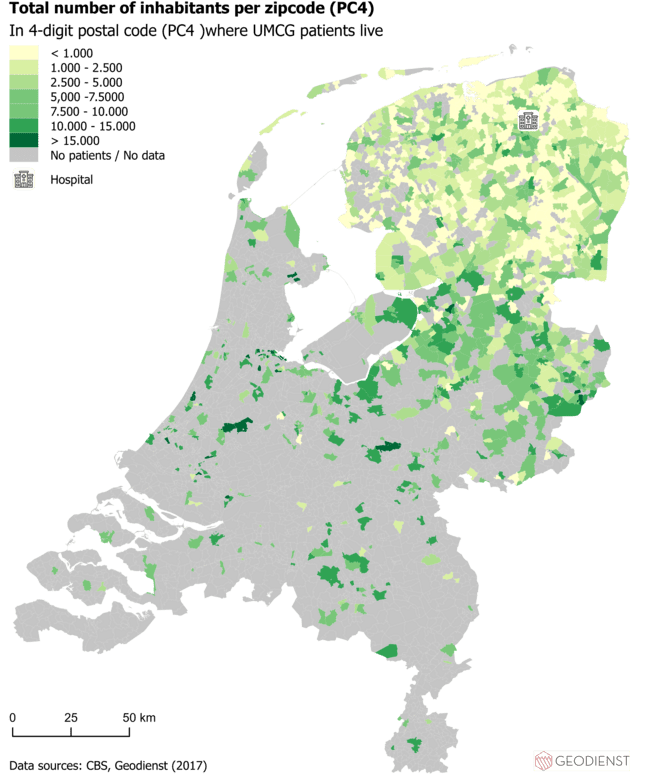


**Figure A10.** The absolute distribution of the general population living in the PC4 areas of the patients.
